# The somatization of structural friction: deciphering the surge of adolescent self-harm in post-transition China

**DOI:** 10.3389/fpsyt.2026.1776994

**Published:** 2026-03-03

**Authors:** Wen-Jing Yan, Qian-Nan Ruan, Dongwu Xu

**Affiliations:** 1 School of Mental Health, Wenzhou Medical University, Wenzhou, China; 2 Wenzhou Depression Specialty Center, Wenzhou Seventh People’s Hospital, Wenzhou, China

**Keywords:** mental health in China, neijuan (involution), non-suicidal self-injury (NSSI), reverse somatic anchoring, structural friction

## Abstract

In the third decade of the 21st century, the mental health landscape of Chinese adolescence has undergone a profound and disturbing transformation. The dominant clinical idiom of distress has shifted from the passive, fatigue-based somatic complaints of “neurasthenia” (shenjing shuairuo) common in the late 20th century, to active, tissue-damaging behaviors, specifically non-suicidal self-injury (NSSI). Recent epidemiological data indicates that NSSI prevalence among Chinese adolescents has risen to levels significantly exceeding global averages, signaling a unique public health crisis that defies standard psychiatric categorization. This perspective article proposes that this surge cannot be adequately explained solely by individual pathology or biological vulnerability. Instead, we suggest it may be more productively understood through the lens of “Structural Friction”, the grinding interface between macro-societal stagnation (characterized by neijuan or involution), high-control family dynamics, and the disembodied nature of digital existence. We propose the “Reverse Somatic Anchoring” model to explain the functional mechanism of NSSI in this specific cultural context. Unlike traditional somatization, where the body passively reflects distress, Reverse Somatic Anchoring suggests that adolescents utilize pain to actively “anchor” a drifting self back into biological reality amidst a crisis of ontological erasure. This article analyzes the tripartite engines of this friction, namely socioeconomic, familial, and technological, and argues that effective intervention requires moving beyond symptom management to address the somatic and structural roots of the crisis.

## 1 Introduction

The clinical presentation of distress among Chinese youth has undergone a radical transformation that mirrors the seismic shifts in the nation’s social fabric. For decades, the dominant idiom of suffering in China was characterized by shenjing shuairuo (neurasthenia), a constellation of passive symptoms such as chronic fatigue, dizziness, and memory loss (1). This diagnosis represented a body wearing down under the collective survival pressures of a developing nation, a passive vessel exhausted by external demands. However, contemporary epidemiological data reveals a violent shift toward active tissue damage. Recent meta-analyses covering the period from 2010 to 2025 indicate that the pooled prevalence of non-suicidal self-injury (NSSI) among Chinese middle school students has reached approximately 22.37% (2), with some regional studies suggesting rates as high as 28.5% in recent years (3).

To interpret this phenomenon, we introduce the concept of “Structural Friction.” We propose that the current epidemic may represent not merely a collection of individual psychiatric disorders but a somatic reaction to the grinding between the relentless demands of the social order, characterized by academic perfectionism, emotional stoicism, and digital performance, and the developmental needs of the biological self. In this article, we propose the Reverse Somatic Anchoring model. While traditional theories view somatization as a passive vessel for repressed emotion (4), we contend that for the modern digital native, NSSI functions as an active mechanism to verify existence. In an era of hollow academic pursuit and digital intangibility, the scar becomes the only undeniable truth. This article will dissect the historical genealogy of this shift, analyze the three engines driving this friction, and propose a new theoretical framework for understanding the utility of pain in the modern Chinese context.

## 2 Historical genealogy: from passive neurasthenia to active laceration

To understand the current crisis, one must contextualize the shift in somatic idioms within the broader history of Chinese psychiatry and society. During the 1980s and 1990s, the psychiatric landscape was dominated by *shenjing shuairuo*, which served as a culturally sanctioned idiom of distress (5, 6). This diagnosis allowed individuals to express suffering arising from rapid social change and political turbulence without incurring the heavy stigma associated with mental illness (7). The neurasthenic body was fundamentally a victim of external forces; it broke down under pressure, eliciting care, rest, and sympathy from the community—a phenomenon known clinically as secondary gain. The remedy for neurasthenia was rest and nutritional replenishment, reinforcing a social contract where the collective acknowledged the individual’s burden.

The transition to NSSI in the 2020s represents a fundamental shift from passive to active somatization, mirroring the transition from a collective economy to a hyper-competitive neoliberal market economy. In the contemporary context, where the individual is viewed as the sole entrepreneur of their own destiny, the passive body is no longer afforded the luxury of weakness. The modern adolescent cannot afford to be neurasthenic because fatigue is viewed as a failure of will. Consequently, the expression of distress has morphed. The self-harming body is not merely failing; it is being aggressively managed by the subject. The act of cutting is an attempt to regulate the self when the external world feels unregulated and overwhelming (8). This mirrors the broader societal shift from the collective unit to the atomized individual. The friction has moved from the interface of the individual and the state to the internal conflict between the individual and the self. The transition from the complaint that “my nerves are weak” to the act of “cutting to feel real” marks a movement from a language of deficit to a language of desperate ontological verification.

It is crucial to emphasize that this genealogical account describes a population-level shift in the dominant idiom of distress rather than a universal trajectory affecting all Chinese adolescents. Contemporary adolescents exhibit substantial heterogeneity in their responses to structural friction. While NSSI has emerged as a prominent pattern, many navigate the same pressures through alternative coping mechanisms, including academic overachievement, social withdrawal, or prosocial peer engagement. Furthermore, neurasthenia and other somatic complaints continue to coexist alongside NSSI, particularly among adolescents from different regional or socioeconomic contexts.

## 3 The engines of structural friction

### 3.1 The socioeconomic engine: involution and policy paradoxes

The defining socioeconomic condition of post-transition China is neijuan (involution), a state of unproductive refinement where increased educational inputs yield diminishing returns (9). For adolescents, this creates a burnout society characterized by what has been termed the Garbage Time of History, where the future feels foreclosed by the endless loop of exam preparation (10). The educational system has transformed into a high-stakes tournament where the only acceptable outcome is total victory, yet the statistical probability of such victory shrinks annually. The somatic consequence of this is a chronic state of hyper-arousal combined with physical exhaustion. The adolescent body is trapped in a “fight or flight” mode without a physical enemy to fight or a place to flee, creating a physiological backlog of stress that requires release.

The “Double Reduction” policy of 2021, while intended to alleviate this burden by banning for-profit tutoring, has inadvertently generated paradoxical second-order effects that have exacerbated the crisis (11). By removing the “third space” of tuition centers—which, despite their academic focus, offered a social environment outside the home—the policy effectively privatized anxiety within the domestic sphere (12). The home has transformed into the primary site of surveillance, and the parent has been deputized as the primary enforcer of academic discipline. This intensification of the home environment directly feeds into family dynamics that are risk factors for NSSI, as the boundary between parent and teacher dissolves. Furthermore, the pressure on school teachers to provide after-school services has led to teacher burnout, further reducing the availability of emotional support within the school system, leaving the adolescent isolated in their struggle (13).

### 3.2 The familial engine: invalidation and alexithymia

The family unit serves as the crucible where structural pressure is transmuted into psychological distress. Research consistently identifies parental psychological control, which refers to intrusive tactics including guilt induction, love withdrawal, and the invalidation of feelings, as a robust predictor of NSSI in Chinese adolescents. In high-pressure environments, this control often manifests as emotional invalidation, where a child’s internal reality is dismissed as incorrect, fragile, or ungrateful (14). When a child expresses sadness or anxiety about academic pressure and is met with responses such as “You have nothing to worry about, we provide everything for you, “ their internal emotional signal is labeled as a malfunction rather than a valid response to stimuli (15).

Persistent invalidation leads to alexithymia, the clinical inability to identify, describe, and process emotions (16). When an adolescent is systematically taught that their verbal encoding of distress is invalid, they lose the linguistic bridge to their own interiority. They feel bad, but they possess no words that are accepted by their caregivers to describe why. In this context, NSSI functions as a translation device. It transmutes abstract, unnamed, and invalid psychological tension into concrete, undeniable, and valid physical pain (17, 18). The blood serves as visual proof of suffering that language failed to convey. It resolves the ambiguity of alexithymia through a pre-verbal, high-fidelity signal. The wound may function as a ‘truth’ that the parent cannot easily dismiss, potentially compelling an acknowledgement of distress that words could not achieve.

### 3.3 The technological engine: digital disembodiment

The post-transition adolescent exists in a state of digital disembodiment. With social life, entertainment, and identity formation occurring largely in virtual spaces such as WeChat, Douyin, and gaming platforms, the physical body is rendered largely inert. It becomes merely a vessel to carry the head from one screen to another. Internet addiction, which correlates strongly with NSSI (19, 20), exacerbates a sense of unreality or floating. The digital world simulates reality but does not offer the resistance of reality; it is frictionless, editable, and impermanent. This lack of physical feedback leads to a terrifying sense of ontological drift, where the adolescent questions the solidity of their own existence (21).

In this context, the Reverse Somatic Anchoring model diverges from standard tension-reduction theories. NSSI is not solely an attempt to escape pain (anxiolysis) but frequently an attempt to escape numbness. The sharp, stinging sensation of injury provides an immediate reality check, re-anchoring the drifting consciousness into the biological housing of the body. Pain possesses a unique ontological status because it is impossible to doubt pain when one is feeling it (22). It is the one sensation that cannot be simulated or scrolled past. Thus, the scar becomes a permanent, analog record in a world of fleeting, deletable digital data. It is an act of reclaiming the body from the virtual ether and asserting, “I exist, and I bleed.”

## 4 The mechanism of reverse somatic anchoring

Before elaborating on the mechanism, we first clarify the epistemological status and operational boundaries of our two core constructs. “Structural Friction” is proposed as a mid-range sociological construct that serves as a macro-level explanatory framework. It identifies the specific societal conditions—namely the grinding interface between neijuan-driven competition, high-control family systems, and digital disembodiment—that generate psychological and somatic distress in contemporary Chinese adolescents. This construct functions at the level of social causation, explaining why this particular cohort, in this particular historical moment, experiences elevated rates of NSSI. It is not intended as a universal theory of self-harm, but rather as a historically and culturally situated explanatory model.

In contrast, “Reverse Somatic Anchoring” operates primarily as a phenomenological and functional mechanism that describes the individual-level process through which NSSI acquires meaning and utility for the adolescent subject. While Structural Friction identifies the external conditions that create distress, Reverse Somatic Anchoring explains the internal logic and subjective experience that makes NSSI a compelling behavioral response. It describes the phenomenological shift from ontological drift (feeling unreal) to somatic verification (feeling real through pain). This construct functions at the level of lived experience and psychological mechanism, addressing the question of how and why the individual engages in self-harm as a solution to existential numbness.

The relationship between these constructs is hierarchical and complementary: Structural Friction creates the conditions of possibility (the macro-context of distress), while Reverse Somatic Anchoring describes the micromechanism of response (the individual’s functional use of pain). Together, they form a multi-level explanatory framework that bridges sociological analysis with phenomenological psychiatry, avoiding both sociological reductionism (which ignores subjective experience) and psychological reductionism (which ignores structural determinants).

Based on the synthesized evidence, we hypothesize that ‘Reverse Somatic Anchoring’ may operate as a cyclical mechanism driven by the intersection of the engines described above. The cycle begins with a pre-condition of unreality, where the adolescent experiences life as performative and intangible due to digital disembodiment and the “hollow” pursuit of *neijuan*. The self feels thinned out, distributed across servers and exam papers, but not “located” in the here and now. This fragile state is then disrupted by a trigger, typically an acute stressor like a poor grade, a peer conflict, or parental criticism. Due to alexithymia and the fear of invalidation, the adolescent cannot verbally process this stress. The emotion is not felt as “sadness” or “anger” but as a “nameless dread” or an unbearable, suffocating numbness.

Consequently, the adolescent engages in the anchoring event of NSSI. This act serves a dual purpose. Neurobiologically, the tissue damage triggers the “pain offset relief” mechanism, releasing endogenous opioids that lower physiological arousal and reduce the backlog of stress (23). Phenomenologically, the sight of blood and sensation of pain pierce the dissociation, allowing the adolescent to feel “real” again (24). The cycle concludes with temporary stabilization, where the subject feels calm, reclaimed from the digital ether, and physically present. However, because the structural friction defined by academic pressure, family dynamics, and digital isolation remains unchanged, the drift inevitably returns, necessitating a repetition of the cycle. This mechanism highlights a darkening social contract; unlike the neurasthenic patient seeking care, the self-harming adolescent seeks control and confirmation of existence in a “last territory” of agency.

## 5 Discussion

### 5.1 Limitations of current approaches

The analysis suggests that while current therapeutic approaches in China (including pharmacotherapy and traditional Cognitive Behavioral Therapy) remain foundational for acute stabilization and symptom management, they may be insufficient in isolation if they fail to address the somatic and structural roots of the behavior. We do not advocate for the abandonment of these established modalities. Rather, we argue that CBT’s focus on correcting cognitive distortions may need to be supplemented when treating alexithymic adolescents for whom the core issue is not a “wrong thought” but a missing somatic connection (25). Treating NSSI merely as a symptom of depression to be medicated misses the functional aspect of the behavior as an anchoring mechanism. Therefore, we propose an integrative approach. If the “anchor” is removed (by preventing self-harm) without reducing the “drift” caused by structural friction, the patient is left defenseless against existential numbness.

### 5.2 Future directions and recommendations

Addressing this crisis requires a paradigm shift from symptom management to structural and somatic re-engagement. First, effective intervention must prioritize somatic therapies that focus on interoceptive awareness. Approaches such as Feel-Own-Move (FOM) are critical for helping adolescents learn to inhabit their bodies through movement, breath, and sensation, rather than through pain (26). The goal is to help the patient feel real without the necessity of tissue damage. Therapies must move beyond the talking cure to include the feeling cure, re-establishing the connection between the mind and the physical self in a non-destructive way.

Simultaneously, interventions must target the family engine through validation training. Parents need training not just in general communication, but specifically in the mechanism of validation, which involves learning to acknowledge their child’s internal reality as real even if they disagree with it (27). This is essential to break the cycle of invalidation that leads to alexithymia. Finally, the concept of digital dieting should be reframed as physical re-engagement. Reducing digital immersion is not merely about limiting screen time but about increasing ontological weight. Physical activities that involve resistance and tangible consequences, such as strength training, craftsmanship, or team sports, can provide the reality check that the digital world lacks (28). These activities offer a good pain of exertion and a visible result of effort, potentially replacing the need for the sharp reality of the blade.

Furthermore, while this framework is derived from the specific sociocultural dynamics of post-transition China, the components of Structural Friction (economic stagnation, high-stakes testing, and digital disembodiment) are increasingly global phenomena. Future research should examine the applicability of the Reverse Somatic Anchoring model in other hyper-competitive East Asian contexts (e.g., South Korea, Japan) as well as in Western settings where digital immersion is reshaping adolescent ontology. Comparative studies could investigate how the specific “flavor” of structural friction varies across cultures and whether the somatic response differs in individualistic versus collectivistic societies, thereby validating the cross-cultural relevance of this construct.

## 6 Conclusion

The precipitous surge of adolescent self-harm in post-transition China represents more than a psychiatric anomaly or a contagion of trends; it serves as a somatic manifestation of the current structural configuration. The Reverse Somatic Anchoring model suggests that when societal friction erodes the subjective sense of self, and when digital existence renders life intangible, the physical body becomes a critical anchor. The existential void associated with neijuan may be countered by pain because pain offers a sensation that feels undeniably real in a world of performative excellence and digital drift. The behavior of self-harm communicates a distress that remains otherwise unarticulated, a signal of limits, of fragility, and of the need for a reality that does not demand the erasure of the self. Addressing this crisis requires more than policy adjustments; it requires a fundamental re-evaluation of how agency, emotion, and reality are constructed for Chinese youth.

## Data Availability

The original contributions presented in the study are included in the article/supplementary material. Further inquiries can be directed to the corresponding author.
